# Different bisphenols induce non-monotonous changes in miRNA expression and LINE-1 methylation in two cell lines

**DOI:** 10.1093/eep/dvab011

**Published:** 2021-11-25

**Authors:** Julia Oldenburg, Maria Fürhacker, Christina Hartmann, Philipp Steinbichl, Rojin Banaderakhshan, Alexander Haslberger

**Affiliations:** Department of Nutritional Sciences, University of Vienna, Althanstraße 14 (UZA II), Vienna 1090, Austria; Department of WAU, Institute of Sanitary Engineering and Water Pollution Control, University of Natural Resources and Life Sciences Vienna, Muthgasse 18, Vienna 1190, Austria; Environment Agency Austria, Spittelauer Lände 5, Vienna 1090, Austria; Environment Agency Austria, Spittelauer Lände 5, Vienna 1090, Austria; Department of WAU, Institute of Sanitary Engineering and Water Pollution Control, University of Natural Resources and Life Sciences Vienna, Muthgasse 18, Vienna 1190, Austria; Department of Nutritional Sciences, University of Vienna, Althanstraße 14 (UZA II), Vienna 1090, Austria

**Keywords:** epigenetic effects, miRNAs, *LINE-1* methylation, bisphenols, BPA, BPS, BPF, *p,p*ʹ-oxybisphenol, BPA β-d-glucuronide, HLF, Caco-2 cells

## Abstract

4,4ʹ-Isopropylidenediphenol (bisphenol A, BPA), a chemical substance that is widely used mainly as a monomer in the production of polycarbonates, in epoxy resins, and in thermal papers, is suspected to cause epigenetic modifications with potentially toxic consequences. Due to its negative health effects, BPA is banned in several products and is replaced by other bisphenols such as bisphenol S and bisphenol F. The present study examined the effects of BPA, bisphenol S, bisphenol F, *p*,*p*ʹ-oxybisphenol, and the BPA metabolite BPA β-d-glucuronide on the expression of a set of microRNAs (miRNAs) as well as *long interspersed nuclear element-1* methylation in human lung fibroblast and Caco-2 cells. The results demonstrated a significant modulation of the expression of different miRNAs in both cell lines including miR-24, miR-155, miR-21, and miR-146a, known for their regulatory functions of cell cycle, metabolism, and inflammation. At concentrations between 0.001 and 10 µg/ml, especially the data of miR-155 and miR-24 displayed non-monotonous and often significant dose–response curves that were U- or bell-shaped for different substances. Additionally, BPA β-d-glucuronide also exerted significant changes in the miRNA expression. miRNA prediction analysis indicated effects on multiple molecular pathways with relevance for toxicity. Besides, *long interspersed nuclear element-1* methylation, a marker for the global DNA methylation status, was significantly modulated by two concentrations of BPA and *p*,*p*ʹ-oxybisphenol. This pilot study suggests that various bisphenols, including BPA β-d-glucuronide, affect epigenetic mechanisms, especially miRNAs. These results should stimulate extended toxicological studies of multiple bisphenols and a potential use of miRNAs as markers.

## Highlights

1. Bisphenol A (BPA), its major metabolite BPA β-d-glucuronide, BPF, BPS, and *p*,*p*ʹ-oxybisphenol are epigenetically active in HLF and Caco-2 cells.
2. Different bisphenols showed changes in the miRNA expression (miR-21, miR-24, miR-146, and miR-155) and *LINE-1* methylation at concentrations between 0.001 and 10 µg/ml in the present pilot study.
3. The results often displayed non-monotonous dose–response relationships and were bisphenol, concentration, and cell-type specific.
4. The expression of miR-155 and miR-24 was most affected by the analyzed bisphenols, revealing U- or bell-shaped curves for most substances.
5. miRNAs could be promising markers for the bioactivity and toxicity of bisphenols.

## Introduction

As the epigenome constitutes the interface between the environment and the genome, epigenetic dysregulation by environmental stressors is likely to disrupt different cellular processes that can contribute to cancer risk [1]. Many environmental chemicals such as bisphenol A (BPA) can act as endocrine-disrupting chemicals (EDCs), thereby impeding estrogenic activity and disrupting neurobehavioral development while stimulating carcinogenicity [2]. Furthermore, BPA was shown to cause epigenetic changes in various research models [3, 4], including non-monotonous dose–response relationships [5]. Non-monotonic dose–response curves (NMDRCs) are defined mathematically by a response where the slope of the curve changes sign from positive to negative, or vice versa, somewhere along the range of doses examined. In this case, the slope of the curve was not identified, but significant changes between the reference and investigated concentrations were assigned to verify non-monotonicity.

As a dynamic regulatory network, the epigenome mediates cellular, tissue-dependent, and thus organismal responses to various stressors from the environment [1]. Epigenetic changes are heritable and lead to adjustments in the gene activity and expression without changing the DNA sequence [6]. These mechanisms encompass DNA methylation and histone modifications as well as non-coding RNAs including microRNAs (miRNAs), among others [7]. DNA methylation depicts the addition of a methyl group to a cytosine of a cytosine–guanine dinucleotide (CpG) site. In promotor regions, this reaction generally results in a suppressed binding activity of transcription factors to the related genes [8]. To estimate global DNA methylation levels, repetitive non-coding elements can be examined [9, 10]. Due to their potential to retrotranspose, a loss of methylation at these sites may lead to chromosomal rearrangements that can contribute to genomic instability and cancer development [11]. *Long interspersed nuclear element-1 (LINE-1)* is a mobile element comprising 17% of the human genome and is thus discussed to indicate global methylation status and DNA stability [12].

miRNAs regulate post-transcriptional gene expression and play an important role in RNA silencing [13]. Therefore, they have critical functions in fine-tuning a wide array of biological processes including cellular differentiation, metabolism, homeostasis, and apoptosis, and some can seriously influence carcinogenesis [14]. On the one hand, similar to that of protein-coding genes, the miRNA expression is regulated by multiple transcriptional networks as well as the epigenetic machinery. On the other hand, miRNAs can themselves repress key enzymes that drive epigenetic remodeling, generating regulatory circuits that have a significant effect on the transcriptional landscape of the cell [15]. Recent evidence also suggests that miRNAs can directly modulate gene transcription in the nucleus through the recognition of specific target sites in promoter regions [15].

As miRNA profiles are surprisingly informative [16], studies point toward the fact that they may be used as biomarkers for toxicity or human diseases [17, 18]. A recent systematic review reported that miRNA dysregulation by BPA can lead to serious health concerns by impeding numerous cellular signaling pathways even at low levels [19]. Furthermore, they have shown that miR-21 was the most commonly impacted miRNA [19]. miR-21 is known for its oncogenic and antiapoptotic activity and its overexpression in multiple solid tumors [15]. Likewise, the upregulation of miR-24 has been associated with poor cancer survival in a recent meta-analysis [20] and is related to apoptosis [21–23]. Target analyses of the most commonly altered gene expressions upon changes in miR-146a showed genes related to cancer and inflammatory and neural diseases [24]. Environmental chemicals such as air pollutants, heavy metals, and BPA have also been reported to stimulate *LINE-1* hypomethylation [25], and *LINE-1* activity is associated with various tumors predominantly found in the lung and colon [26]. Following BPA treatment in Michigan Cancer Foundation-7 (MCF-7) breast cancer cells, a study revealed a reduced methylation level of oncogenes and an increased methylation of tumor-suppressor genes [27].

Consequently, the accurate quantification of epigenetic effects is not only crucial to clinical diagnosis, drug development, and cancer treatment [28], but also to the authorization of chemicals. BPA is an environmentally omnipresent EDC that can enter the body via the skin, the respiratory system, and the gastrointestinal tract [19]. For example, due to different adverse health effects such as effects on reproduction, disruption of the endocrine system and neurobehavioral development, epigenetic effects, or immune disorders [3, 29], BPA is included in the candidate list of substances of very high concern according to Article 59 [10] of the Regulation (EU) No 1907/2006 (REACH) since 2017 [30] and also regulated in products such as baby bottles made of polycarbonate (Regulation (EU) 10/2011, amended by Regulation (EU) 2018/213), thermal paper (Regulation (EU) 2016/2235), and cosmetics (Regulation (EU) 1223/2009, latest amended by Regulation (EU) 2020/1684). Therefore, BPA will be replaced, but the question is: for other bisphenols (BPS, BPF, BPAF, etc.) with similar properties—do they also have similar effects? A systematic review revealed that the potential of BPS and BPF to interfere with the hormonal system is comparable with the activity of BPA [31]. Accordingly, there is concern about the safety of other bisphenols. However, scarcely any report has focused on their epigenetic effects as well as on BPA’s major metabolite BPA β-d-glucuronide until now. Therefore, this pilot study aimed to examine the impact of various bisphenols on miRNA and methylation patterns in human Caco-2 and human lung fibroblast (HLF) cell lines. To analyze physiologically relevant concentrations to high doses of bisphenols and to consider NMDRCs, concentrations of 1 ng/ml–10 µg/ml were administered. As the various bisphenols have different water solubilities and could be adsorbed by plastic materials [32, 33], the actual concentrations were measured in the cell media before and after incubation.

## Materials and Methods

### Chemicals and Analytical Standards

Bisphenols were purchased in chemically pure quality from Sigma-Aldrich (BPA, BPS), Tokyo Chemical Industry (BPF), Accela (*p*,*p*ʹ-oxybisphenol), and Santa Cruz Biotechnology (BPA β-d-glucuronide). Stock solutions were prepared from solid standards in concentrations of 10 mg/ml bisphenol in ethanol (EtOH) and were dissolved in cell culture medium to the respective concentrations.

### Cell Culture and Treatment

Caco-2 cells and HLFs were kindly provided by Prof. Dr Walter Jäger, Department of Pharmaceutical Sciences, University of Vienna and Prof. Dr Siegfried Knasmüller, Institute of Cancer Research, Medical University of Vienna. These cell lines were chosen to imitate gastrointestinal and lung exposure. Cells were stored at −70°C in liquid nitrogen before thawing and then incubated in 25 or 75 cm^2^ flasks at 37°C in a humidified atmosphere with 5% CO_2_. Caco-2 cells were cultured in Dulbecco’s modified Eagle’s medium (DMEM, high glucose; Sigma-Aldrich), whereas the medium for HLFs consisted of RPMI 1640 (Thermo Fisher). Both media were supplemented with 1% (v/v) penicillin/streptomycin (Invitrogen) and 10% (v/v) fetal bovine serum (Merck). Cells were passaged at 70–80% confluency after two to three days. Prewarmed phosphate-buffered saline (PBS, HyClone™) and Accutase® (Biowest) solution were used to rinse the cell monolayer and for subsequent cell detachment.

**Figure 1:**
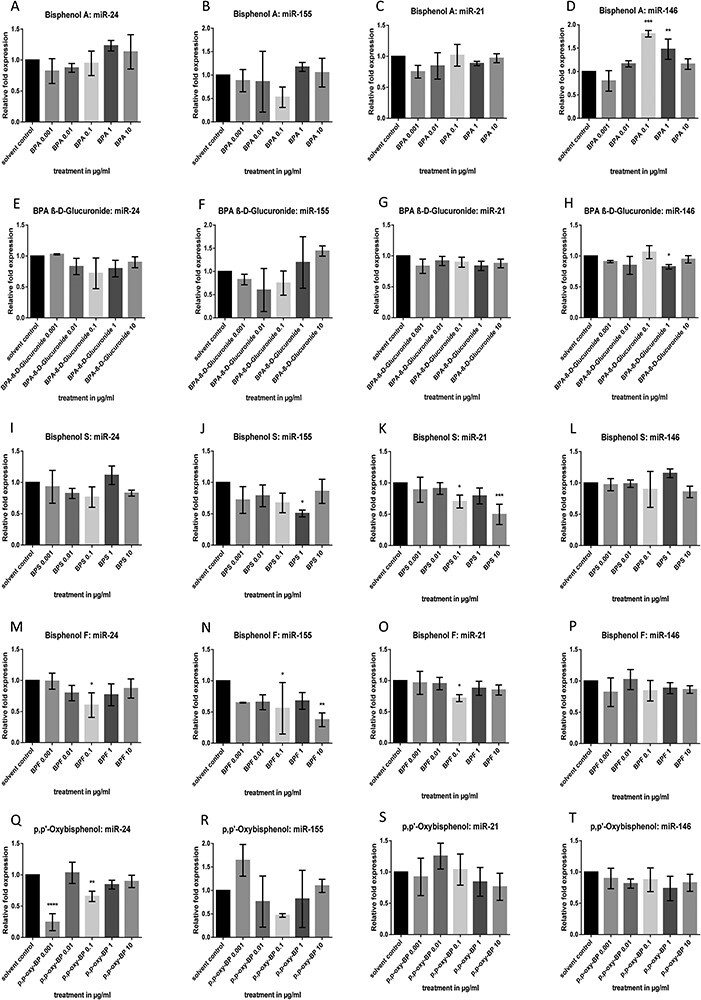
Effects of BPA, BPA β-d-glucuronide, BPS, BPF, and *p*,*p*ʹ-oxybisphenol on miR-24, miR-155, miR-21, and miR-146a expression levels in Caco-2 cells. Cells were exposed to 0.001, 0.01, 0.1, 1, and 10 μg/ml of the respective bisphenol for 24 h, and the miRNA expressions were detected by qRT-PCR. Data are shown as the mean of relative fold changes ± SD of a minimum of three replicates per treatment

**Figure 2:**
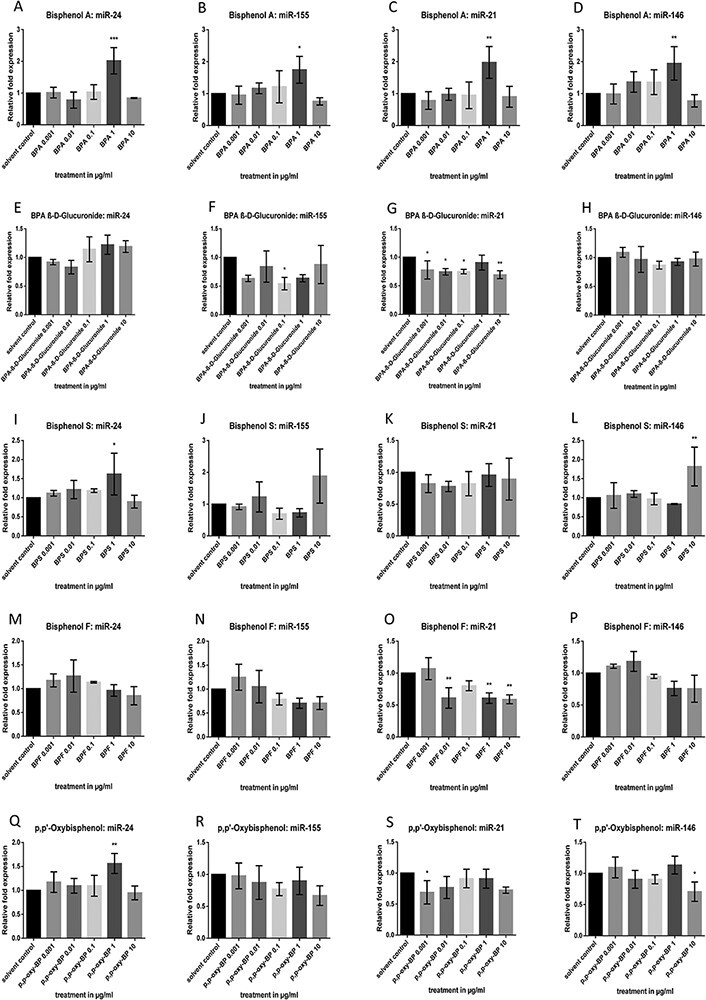
Impact of BPA, BPA β-d-glucuronide, BPF, BPS, and *p*,*p*ʹ-oxybisphenol on miR-24 and miR-155, miR-21, and miR-146a expression levels in HLFs. Cells were treated with 0.001, 0.01, 0.1, 1, and 10 μg/ml of the respective bisphenols for 24 h, and the miRNA expressions were detected by qRT-PCR. Data are shown as the mean of relative fold changes ± SD of three replicates per treatment

**Figure 3:**
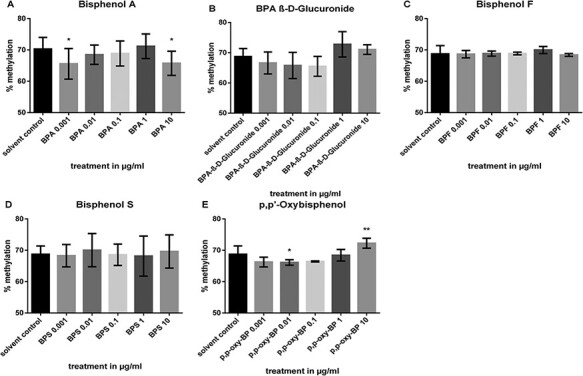
*LINE-1* methylation levels of Caco-2 cells in response to various concentrations of BPA, BPA β-d-glucuronide, BPF, BPS, and *p*,*p*ʹ-oxybisphenol. Cells were treated with 0.001, 0.01, 0.1, 1, and 10 μg/ml of the respective bisphenol for 24 h, and the methylation analysis was performed by HRM. Data are shown as the mean ± SD of a minimum of three replicates per treatment

The cells were seeded at a density of 40 K cells/cm^2^ in 24-well plates and were incubated for two to three days at 37°C in an incubator with 5% CO_2_. Cell cultures were treated with 0.001, 0.01, 0.1, 1, and 10 μg/ml of BPA, BPA β-d-glucuronide, BPS, BPF, and *p*,*p*ʹ-oxybisphenol for 24 h. Untreated cell cultures were used as controls. In addition, EtOH (0.01% (v/v)) was chosen as the respective solvent control (indicated in the graphs (Figs 1–3) and used for normalization) since no significant differences were found between the mean value of the EtOH (0.01% and 0.1% (v/v)) and the untreated controls.

*P < 0.05; **P < 0.01; ***P < 0.001; ****P < 0.0001 compared to the solvent control

*P < 0.05; **P < 0.01; ***P < 0.001; and ****P < 0.0001 compared to the solvent control. Abbreviations: BP, bisphenol

*P < 0.05; and **P < 0.01 compared to the solvent control. Abbreviations: BP, bisphenol

### RNA Extraction and Quantitative RT-PCR Analysis

After treatment, the total RNA of the cells was isolated using miRNeasy Micro Kit (Qiagen) as per the manufacturer’s instructions for cell culture. RNA quantity and quality were assessed with a Nanodrop One spectrophotometer (Thermo Scientific). Subsequent reverse transcription and cDNA amplification were undertaken according to the protocol for TaqMan™ Advanced miRNA cDNA Synthesis Kit (Thermo Fisher) and obtained cDNA was stored at −20°C. Quantitative real-time PCR was performed using TaqMan™ Fast Advanced Master Mix and TaqMan™ Gene Expression Assays (Thermo Fisher) for miR-24-3p, miR-155-5p, miR-21-5p, and miR-146a-5p according to the user guide supplied by Thermo Fisher. To calculate the miRNA relative fold changes, the ΔΔCt method was used with miR-93 as an endogenous control. This stable internal reference has recently been recommended for human cancer cell lines and plasma samples [34, 35].

### DNA Extraction, Bisulfite Conversion, and DNA Methylation Analysis

Global DNA was extracted from the cells using the QIAamp DNA Mini Kit (Qiagen) according to the manufacturer’s protocol for cell culture. DNA samples were stored at −20°C before the bisulfite conversion was performed. To convert unmethylated cytosines to uracil and thus to distinguish between the latter and unaltered 5-methylcytosines, bisulfite treatment was carried out using the Qiagen EpiTect Bisulfite Kit (Qiagen) as explained in the manufacturer’s instructions. DNA concentrations were determined with a Nanodrop One (Thermo Fisher Scientific) spectrophotometer. *LINE-1* promoter methylation was assessed by methylation-sensitive high-resolution melting (HRM) curve analysis. Each reaction was performed in technical duplicates using the EpiTect HRM PCR Kit (Qiagen) according to the EpiTect® HRMTM PCR handbook. Methylation standards of 0% and 100% were mixed to obtain 0, 25, 50, 75, and 100% methylated samples and were included in the assay. PCR amplification and HRM analysis were performed with a Rotor-Gene® Q (Qiagen) containing a 72-well rotor. The data were analyzed with Rotor-Gene® Series Software Version 2.3.1 that produced normalized melting curves. After exporting relative fluorescence units to GraphPad Prism (Version 6), the area under the curve was calculated and linear regression was applied to interpolate each sample value from the data of the standard curve.

### Chemical Analysis

The supernatant of the cell cultures (approximately 800 µl) was transferred into 1.5 ml centrifuge tubes and stored at −18°C until analysis at the Environmental Agency Austria. For sample preparation, the supernatants were centrifuged (10 min, 17 000 rpm) to separate the solid matrix from the liquid phase. In total, 200 µl of the supernatant were spiked with an isotope labeled standard (BPA Ring d8) and analyzed using a Ultra High Performance Liquid Chromatography system coupled to triple quadrupole tandem mass spectrometry (Waters Aquity I Class and Waters Xevo TQ-S) in negative electrospray ionization mode. Chromatographic separation was performed on a reversed-phase column. Detection of the different bisphenols was conducted with typical fragment ions ([M–H]) for each compound in the multiple reaction mode. Quantification was performed using internal (for bisphenol A) and external calibration curves ranging from 0.5 ng/ml to 100 ng/ml. Higher concentrated supernatants were diluted with HPLC water to reach suitable concentrations. To check for matrix effects all dilution levels of the two cell lines were spiked with the bisphenols and recovery rates of this spiking-trial were considered in the calculation of the results.

### Statistical Analysis

All experiments were performed at least in triplicates, and the results are presented as the mean of each treatment with standard deviation (SD) of the mean. Subsequent data analysis and presentation were done using GraphPad Prism software (Version 6). Data were subjected to analysis of variance, and the Sidak test for multiple comparisons was used to separate the means that were considered statistically significant. Statistical significance was set to a *P*-value of ≤ 0.05.

### MiRNA Target Prediction Analysis

The application miRNet (www.mirnet.ca/) was used to predict target genes and networks of miR-24-3p, miR-155-5p, miR-21-5p, and miR-146a-5p. The obtained non-tissue-specific gene targets were classified into functional groups with an integrated device in miRNet that makes use of miRTarBase Version 8.0 database and the Kyoto Encyclopedia of Genes and Genomes (KEGG) pathways. The most frequent cellular functions and/or signaling pathways of all four miRNAs as well as specific genes that are regulated by three of the four examined miRNAs were selected for the Results section of this paper.

## Results

### MiRNA Expression in Bisphenol-treated Caco-2 Cells

Caco-2 cells and the HLF cell line were used to analyze the epigenetic effects of bisphenols. After incubation of Caco-2 cells with bisphenols for 24 h, miRNA levels of BPA-responsive miRNAs were evaluated. Based on preliminary experiments with BPA and estrogen analyzing various miRNAs (including miR-19b, miR-21, miR-27b, miR-122, miR146a, miR-155, and miR-205), miR-24, miR-155, miR-21, and miR-146a were selected for further analysis (Fig. 1A–T), as they showed the most promising effects. A significant upregulation of miR-146a (Fig. 1D) was observed after exposure of 0.1 and 1 µg/ml BPA (*P* < 0.001, *P* < 0.01, respectively) versus the value of the EtOH control. The bar graph suggests a concentration-dependent bell-shaped curve. BPA β-d-glucuronide significantly downregulated the expression of miR-21 (Fig. 1H) at a concentration of 1 µg/ml (*P* < 0.05) as compared with the control value. In addition, a significantly lower miR-21 level (Fig. 1K) was observed with 0.1 and 10 µg/ml BPS (*P* < 0.05 and *P* < 0.001, respectively), whereas BPS attenuated miR-155 expression (Fig. 1J) at a concentration of 1 µg/ml (*P* < 0.05). BPF treatment points towards a U-formed dose–response curve for miR-24 (Fig. 1M) with a significantly reduced miRNA level at 0.1 µg/ml (*P* < 0.05). Incubation with 0.1 µg/ml BPF also lowered the levels of miR-21 (Fig. 1O). Likewise, miR-155 expression (Fig. 1N) was generally diminished after BPF exposure leading to significant downregulations at concentrations of 0.1 and 10 µg/ml BPF (*P* < 0.05 and *P* < 0.01, respectively) compared with the solvent control. *p*,*p*ʹ-Oxybisphenol significantly reduced miR-24 (Fig. 1T) levels at low doses (0.001 and 0.1 µg/ml: *P* < 0.0001 and *P* < 0.01, respectively) in comparison with the EtOH control.

### MiRNA Expression in Bisphenol-treated HLFs

After analyzing the effects of bisphenol compounds on miRNA profiles in Caco-2 cells, the same 24 h experiment was carried out using HLFs (Fig. 2A–T). Following BPA exposure, we observed a significant upregulation of miR-24 (Fig. 2A), miR-155 (Fig. 2B), miR-21 (Fig. 2C), and miR-146 (Fig. 2D) levels with 1 µg/ml BPA (*P* < 0.05) when compared with the value of the EtOH control. Metabolite BPA β-d-glucuronide significantly reduced the expressions of miR-155 (Fig. 2F) at 0.1 µg/ml (*P* < 0.05) and of miR-21 at 0.001, 0.01, 0.1, and 10 µg/ml (*P* < 0.05) as compared with the solvent control. Regarding BPS, a concentration of 1 µg/ml (Fig. 2I) led to a significantly elevated miR-24 expression (*P* < 0.05) and enhanced miR-146 levels (Fig. 2L) at 10 µg/ml (*P* < 0.01) versus the EtOH control. In contrast to this, significantly reduced levels of miR-21 (Fig. 2O) were observed with 0.01, 1, and 10 µg/ml BPF (*P* < 0.01) compared with the EtOH solvent control. *p*,*p*ʹ-Oxybisphenol triggered an enhanced expression of miR-24 (Fig. 2Q) at a concentration of 1 µg/ml (*P* < 0.01) and reduced the levels of miR-21 (Fig. 2S) at 0.001 µg/ml (*P* < 0.05) and miR-146 (Fig. 2T) and 10 µg/ml (*P* < 0.05).

### *LINE-1* Methylation Level in Bisphenol-treated Caco-2 Cells

To evaluate potential modulations in *LINE-1* promoter methylation, Caco-2 cells were exposed to increasing concentrations of bisphenols for 24 h. BPA (Fig. 3A) decreased the methylation level at the lowest and the highest concentration observed (0.001 and 10 µg/ml, *P* < 0.05) suggesting a bell-shaped dose–response relationship. Likewise, *p*,*p*ʹ-oxybisphenol (Fig. 3E) reduced the methylation level of the *LINE-1* promotor region at a concentration of 0.01 μg/ml (*P* < 0.05), while 10 μg/ml led to a small increase (*P* < 0.01) contra the solvent control, thus revealing an NMDR.

### Analysis of the Bisphenol Recovery Rates in the Cell Supernatants

The measurement of BPA in the cell media supernatants showed good recovery rates related to the theoretically estimated concentrations (97.8% ± 5% for Caco-2 cells; 104% ± 9% for HLFs). Preliminary results showed higher deviation for other bisphenols (e.g. *p*,*p*ʹ-oxybisphenol) (not lower than a recovery of 80%). To check matrix effects all dilution levels of the two cell lines were spiked with the bisphenols and recovery rates were calculated. Some bisphenols (e.g. BPS or *p*,*p*ʹ-oxybisphenol) showed a higher influence of matrix effects during the measurement, other bisphenols (e.g. BPF) were not influenced in a significant way during the measurement. Recovery rates determined from the spiking trial were considered in the calculation of the final concentrations. Other effects that may have influences such as adhesive effects of bisphenols to cell-particles could not be investigated during this study. The determination of the real concentrations of BPA β-d-glucuronide was not possible due to strong matrix effects caused by the cell media.

### Potential Gene Targets of Selected miRNAs: Networks Analysis and Functional Pathways

In order to elucidate possible molecular mechanisms of bisphenol-induced toxicity, the miRNA network tool miRNet was used to explore miRNA target genes and the related signaling pathways and cellular functions. Pathway analysis of miR-24, miR-155, miR-21, and miR-146a classified their target genes and the most enriched and significantly associated biological functions can be seen in Table 1. Cancer- and immune system-related cell signaling pathways, other signal transduction pathways, and genes associated with focal adhesion, the cell cycle, RNA transport, and apoptosis were among the top-ranked pathways. Moreover, Table 2 shows the predicted target genes of the selected miRNAs that were identified to be regulated by at least three out of these four miRNAs.

**Table 1:** Predicted signaling pathways and functions of miR-24, miR-155, miR-21, and miR-146a target genes. Gene network analysis was done using miRNet and showed the most common related KEGG pathways

Gene pathways and functions	Number of target genes	*P*-value
Pathways in cancer	91	2.4e–10
Mitogen-activated protein kinase signaling pathway	65	0.0000856
Focal adhesion	47	0.00217
Cell cycle	39	0.00000705
RNA transport	38	0.0000271
Toll-like receptor signaling pathway	36	1.81e–7
Wnt signaling pathway	33	0.0138
T-cell receptor signaling pathway	32	0.0000202
p53 signaling pathway	30	2.04e–8
Apoptosis	30	0.00000367
Neurotrophin signaling pathway	30	0.00755
TGF-β signaling pathway	22	0.00885
Erythroblastic leukemia viral oncogene signaling pathway	22	0.0135
B-cell receptor signaling pathway	20	0.00997

**Table 2:** Common target genes of the analyzed miRNAs miR-24, miR-155, miR-21, and miR-146a. Gene pathway analysis revealed that at least three out of four miRNAs regulated the above-mentioned human genes and were identified by miRNet

Gene symbol	Gene name
NFAT5	nuclear factor of activated T cells 5
APAF1	apoptotic peptidase activating factor 1
BRCA1	BRCA1 DNA repair associated
E2F2	E2F transcription factor 2
EGFR	epidermal growth factor receptor
ICAM1	intercellular adhesion molecule
MYC	MYC proto-oncogene, bHLH transcription factor
NFKB1	nuclear factor kappa B subunit 1
OLR1	oxidized low density lipoprotein receptor 1
SP1	Sp1 transcription factor
TGFB1	transforming growth factor beta 1
VHL	von Hippel-Lindau tumor suppressor
RNF11	ring finger protein 11
PLEKHA2	pleckstrin homology domain containing A2
DCAF10	DDB1 and CUL4 associated factor 10
CCND1	cyclin D1
CDC73	cell division cycle 73
ZNF260	zinc finger protein 260

## Discussion

Human studies have reported that even nanomolar exposure concentrations of BPA are associated with an increased risk of metabolic, cardiovascular, and neurological diseases as well as cancers [36, 37]. As a result of these health concerns and a subsequent ban of BPA in baby products and receipts, industries have started to replace BPA with other bisphenol-group compounds such as BPS and to a smaller extent also BPF, although studies have already questioned their safety [31]. Moreover, our research showed that BPA is epigenetically highly active, which possibly links bisphenols exposure to the occurrence of various diseases [4, 38]. Therefore, the goal of our study was to investigate epigenetic effects of BPA, its metabolite BPA β-d-glucuronide, and other bisphenols such as BPS, BPF, and *p*,*p*ʹ-oxybisphenol using miRNA profiling and *LINE-1* methylation analysis. Due to the number of selected bisphenols, a limitation of this study is the small number of replications and tested concentrations. Therefore, we consider the present project as a pilot study. However, to our knowledge, this is one of the first reports analyzing epigenetic responses to different bisphenols and taking into consideration the metabolite BPA β-d-glucuronide in Caco-2 as well as in HLF cells.

In the current study, different bisphenols altered miRNA expressions and *LINE-1* methylation in distinct ways. As a dynamic regulatory network, the epigenome mediates cellular, tissue-dependent, and organismal responses to various stressors from the environment. It is interesting to note that the bar charts of most bisphenol-group compounds revealed NMDRCs that often showed a reduced miRNA expression at the mean concentration of 0.1 µg/ml in Caco-2 cells. NMDRCs can be found in the literature for EDCs and BPA in a variety of biological systems including cultured cells, whole organ cultures, laboratory animals, and humans [5, 39]. Following BPA treatment in Caco-2 cells, the bar charts of not only miR-146, but also of miR-155 (Fig. 1B) tend to display non-monotonous dose–response relationships. Consistently, the impact of BPA β-d-glucuronide on miR-24 (Fig. 1E) and miR-155 levels (Fig. 1F) suggests NMDRCs showing the lowest expression at concentrations of 0.1 and 0.01 µg/ml, respectively. *p*,*p*ʹ-Oxybisphenol treatment also resulted in non-significant NMDRCs regarding miR-155 (Fig. 1R); the lowest miR-155 expression was detected at 0.1 µg/ml, whereas 0.001 µg/ml *p*,*p*ʹ-oxybisphenol tended to elevate miR-155 (Fig. 1R). In HLF experiments, observed dose–response curves seemed less pronounced (e.g. BPF regarding miR-24, miR-155, and miR-146; Fig. 2M, N, and P). Comparable non-monotonous effects could be seen after incubation with BPA as well as BPS (here only regarding miR-24; see Fig. 2I). Moreover, our results indicated that miR-155 was generally most affected by different bisphenols as can be observed by a consistent downregulation in Caco-2 cells.

Regarding *LINE-1* methylation, the data suggest a bell-shaped curve after BPA exposure and a partly U-shaped dose–response curve after treatment with *p*,*p*ʹ-oxybisphenol. Similarly, although non-significant, metabolite BPA β-d-glucuronide (Fig. 3B) tended to attenuate *LINE-1* methylation at the lower concentrations (0.001–0.1 μg/ml) but led to an elevation in the µg/ml range (1 and 10 µg/ml).

Therefore, our data demonstrated that other bisphenols can affect miRNA and methylation levels and that the BPA metabolite BPA β-d-glucuronide is also biologically active with regard to miRNA modulation. BPA is metabolized to BPA β-d-glucuronide in the liver, its predominant metabolite, and excreted via the kidneys [40]. For a long time, research suggested that BPA β-d-glucuronide was inactive due to its inability to bind to estrogen receptors [41]. Nonetheless, it could exert influence via non-estrogenic activity, which was revealed for BPA before (e.g. glucocorticoid receptor) [42]. In human serum and urine samples, BPA β-d-glucuronide was shown to be present at higher concentrations than free BPA (up to 11.9 and 18.7 ng/ml, respectively) [43] and could also be found in human blood [40]. Boucher *et al.* demonstrated that BPA β-d-glucuronide was able to induce adipogenesis in 3T3-L1 cells [44].

It could be observed that the results showed differences between the two cell lines. Higher concentrations of bisphenols in HLFs often resulted in an increased expression of miRNAs. The differentially expressed miRNAs suggest that they were altered individually and not via changes in the miRNA processing machineries. This is supported by a study showing that neither DROSHA nor DICER was modulated upon BPA treatment [45].

MiRNAs play a role in mRNA translation by silencing or degrading mRNA products thereby regulating multiple cellular processes. MiR-155 is enhanced in B-cell lymphomas, breast, lung, colon, and pancreatic tumors and hematopoietic malignancies [15] and is associated with inflammation [46]. Not many studies have evaluated the impact of BPA and its substitutes on miR-155 expression so far. In HA cells (hemangioma: HDEC and CRL-2686 cells), nanomolar concentrations of BPS reduced miR-155 levels and thus triggered an increase of basic fibroblast growth factor expression, which is related to HA development [47]. Similarly, our data displayed a downregulating effect after addition of moderate concentrations of all analyzed bisphenols in Caco-2 cells, whereas higher doses of BPA (1 µg/ml) and BPS (10 µg/ml) in HLFs tended to increase miR-155 expression, likely indicating cell type- and concentration-related differences. An upregulation of miR-155 was also observed in an *in vitro* model of bovine cumulus–oocyte complexes with 50 µg/ml BPA [48] and in a study applying 0.025 µg/ml BPA in human placental cells, but for a period of six days [49].

As a key miRNA of numerous physiological processes such as proliferation and apoptosis, miR-24 was also shown to be overexpressed in pathological conditions such as cancer, multiple sclerosis, hyperglycemia, and Alzheimer’s disease [50]. In our study, the mean concentration (0.1 µg/ml) of BPF and *p*,*p*ʹ-oxybisphenol and other bisphenols significantly decreased, and BPA β-d-glucuronide tended to attenuate miR-24 in Caco-2 cells. In line with these results, Cho *et al.* used microarray analysis to detect differently regulated miRNAs in male testes of mouse Sertoli cell line TM4 and found two-fold lower miR-24 levels after 20 µg/ml BPA exposure for 24 h [51]. In contrast to this, we detected a significant upregulation with BPA, BPS, and *p*,*p*ʹ-oxybisphenol in HLFs, although only at one concentration (1 µg/ml). Again, these differences could possibly be attributed to different doses of BPA and distinct cell types.

BPA was shown to increase miR-146a involved in endocrine regulation and immune homeostasis [52]. Consistent with our BPA results, De Felice *et al.* found a significantly higher expression of miR-146a in placental samples of pregnant women living in areas with a high BPA pollution [24]. As fetuses are undergoing rapid growth and development, prenatal BPA exposure is specifically critical [53]. In human placental cell lines, BPA led to an attenuated proliferation and an elevated sensitivity to the DNA damaging agent bleomycin [49]. Similarly, BPA triggered enhanced miR-146a expression in testicular Leydig cells of mice thereby impeding steroidogenesis via *Metastasis Associated 1 Family Member 3* mRNA repression [52]. Nonetheless, except for an upregulation with 10 µg/ml BPS in HLFs, we did not find significantly induced miR-146a expression levels in response to the other bisphenol compounds, suggesting that this dysregulation might be restricted to BPA. However, the highest concentrations of *p*,*p*ʹ-oxybisphenol decreased miR-146a, although most BPA substitutes did not result in any changes. A stable profile of miR-146a was also found after BPA or BPS addition to bovine oocytes at the LOAEL of 50 µg/ml [48], revealing possible tissue or species-dependent differences.

As a miRNA controlling antiapoptotic regulation, miR-21 is crucial for cell survival by suppressing apoptotic gene expression [54]. Thus, it can be speculated that a partial downregulation observed after BPF, BPA β-d-glucuronide (HLFs), and BPS (Caco-2 cells) in the current study could enhance apoptosis-related transcription. Similar to our results following exposure to certain bisphenols, BPA also reduced the expression level of miR-21 in 3T3-L1 preadipocytes (80 µM) [55] and MCF-7 cells mediated by ER (estrogen receptor)-α [56]. On the other hand, miR-21 is also called an onco-miR due to its inhibiting effect on the tumor-suppressor gene *PTEN* [57], which is supposed to be associated with BPA’s cancer-promoting impact in reproductive tissues [48]. In Caco-2 cells, we saw a slight upregulation following treatment with *p*,*p*ʹ-oxybisphenol. Tilghman *et al.* explained differential effects of BPA on miR-21 in MCF-7 breast cancer cells by ER-positive or -negative signaling pathways [56].

Using miRNet, a miRNA-centered pathway database, we identified numerous gene targets that are connected to modulations in miR-24, miR-155, miR-21, and miR-146a. Pathway enrichment of these miRNAs demonstrated that the most common targets are related to cancer, the mitogen-activated protein kinase signaling pathway, and focal adhesion. Furthermore, changes in the analyzed miRNAs could cause dysregulations in cell cycle progression (e.g. *E2F2*), RNA transport, and apoptosis (e.g. *APAF1*). Likewise, alterations in various signaling pathways were predicted by miRNet including Toll-like receptor (merging into NF-κB activation), T-cell receptor (e.g. *NFAT*), TGF-β, p53, and Wnt signaling pathway. These cascades are mostly linked to general cellular functions such as cell proliferation and differentiation as well as immune responses. Several studies have shown that dysfunctions of these signaling pathways are associated with tumorigenesis [58–60]. In our target analysis, we found genes that were potentially modulated by three out of the four investigated miRNAs (e.g. *TGFB1, MYC, ErbB, BRCA1, EGFR*). Various studies confirm changes in the expression of these genes or proteins upon bisphenol exposure: in breast cancer cell lines, low concentrations of BPAF [61] and tetrachloro-BPA [62] enhanced protein expression of c-Myc and cyclin D1, important for cell cycle progression. In mice, this led to an activation of epidermal growth factor receptor (EGFR) and subsequent increases in growth factor expression related to mammary carcinoma [63]. EGFR, linked to the erythroblastic leukemia viral oncogene signaling pathway, is the receptor for epidermal growth factor and TGF-α, among others, and thus stimulates cell growth while inhibiting apoptosis, which is why it is also considered as an oncogene [64]. In a murine and intestinal epithelial cell model, the transcriptional activity of NF-κB, production of inflammatory cytokines, as well as apoptotic processes were promoted by BPA, explaining the risk for BPA-induced gut injury [65].

Aberrant DNA methylation due to BPA exposure has been reported in various studies [4, 38, 66]. We therefore assessed *LINE-1* methylation in Caco-2 cells, which is a marker of global methylation status [12]. As a common element of the human genome, its hypomethylation is linked to genomic instability and a higher risk of cancer development [26]. Our analysis of cytosine–guanine dinucleotide shores in the *LINE-1* promotor revealed significantly lower methylation following the lowest and highest concentration of BPA and thus showing an NMDRC. Likewise, treatment with low-dose *p*,*p*ʹ-oxybisphenol resulted in diminished methylation, whereas a rise was observed after the addition of the highest concentration. We found a similar trend towards a non-monotonous dose–response effect with BPA β-d-glucuronide, indicating that both—low and high—concentrations can affect DNA methylation. Most other bisphenols resulted in a diminished methylation level, which can result in an enhanced gene expression similar to changes in the miRNA expression. Our results are mostly consistent with Miao *et al.* who found lower methylation levels at *LINE-1* sites in the sperm, but not in the peripheral blood, of occupationally BPA-exposed men in China [25]. Nahar *et al.* showed organ-dependent methylation-modifying effects: hypermethylation of *LINE-1* with BPA was only detected in fetal human placenta [67]. The only study that also examined BPA as well as BPS and BPF found a tendency of decreased *LINE-1* methylation in MCF-7 breast cancer cells [66]. Thus, BPA methylation aberrations may only be visible in certain cell lines or tissues. We thus recommend to further validate the effects and possible mechanisms for differences in the results. Likewise, *LINE-1* methylation is an indicator of global DNA methylation, so that the cytosine–guanine dinucleotide islands of more specific DNA promoters should be explored in future studies.

As can be seen in our results mostly describing NMDRCs, the application of several concentrations is important to show the whole spectrum of effects and to not limit the comparability with other studies. We therefore highly recommend making use of various concentrations in future bisphenol research. Therefore, a strength of our study is not only the application of concentrations that are physiologically and toxicologically relevant but also the use of different bisphenols. Likewise, we investigated the actual BPA concentrations in the cell suspensions by UHPLC and found good recovery rates.

## Conclusion

Aiming to study epigenetic effects of BPA in comparison with other bisphenols, we explored miRNA profiles and *LINE-1* methylation after treatment with various concentrations of BPA, its major metabolite BPA β-d-glucuronide, BPF, BPS, and *p*,*p*ʹ-oxybisphenol. Bisphenols are discussed to affect cell growth, cell invasion, apoptosis, and other cellular processes and miRNAs contribute to their regulation. Therefore, miRNAs might belong to biomarkers for the bioactivity of bisphenols. Although being preliminary, the results showed that miRNA and methylation patterns were deregulated by different bisphenols, which often displayed non-monotonous dose–response curves. These effects were not only cell-type specific but also highly individual concerning the selected bisphenols. Changes in the expression of essential miRNAs could also be a new approach to study possible toxic epigenetic consequences of bisphenols and the metabolite. Furthermore, not only the effects on the molecular targets of miRNAs need to be further investigated, but also the epigenetic activity of BPA β-d-glucuronide should be discussed for its toxicological relevance in future studies.

## Supplementary Material

dvab011_Supp

## References

[1] Herceg Z , Vaissière T. Epigenetic mechanisms and cancer: an interface between the environment and the genome. *Epigenetics* 2011;6:804–19. 21758002 10.4161/epi.6.7.16262

[2] Mustieles V , D’Cruz SC, Couderq S et al. Bisphenol A and its analogues: a comprehensive review to identify and prioritize effect biomarkers for human biomonitoring. *Environ Int* 2020;144:105811. 10.1016/j.envint.2020.105811 32866736

[3] Cariati F , Carbone L, Conforti A et al. Bisphenol A-induced epigenetic changes and its effects on the male reproductive system. *Front Endocrinol (Lausanne)* 2020;11:453. 10.3389/fendo.2020.00453 PMC7406566 32849263

[4] Camacho L , Pogribny IP. Epigenetic effects of bisphenol A (BPA): a literature review in the context of human dietary exposure. In: Patel V and Preedy V (eds.). *Handbook of Nutrition, Diet, and Epigenetics*, Cham (Switzerland): Springer, 2017, 1–20.

[5] Vandenberg LN . Non-monotonic dose responses in studies of endocrine disrupting chemicals: bisphenol A as a case study. *Dose Res* 2014;12:259–76. 10.2203/dose-response.13-020.Vandenberg PMC4036398 24910584

[6] Wetherill YB , Akingbemi BT, Kanno J et al. In vitro molecular mechanisms of bisphenol a action. *Reprod Toxicol* 2007;24:178–98. 17628395 10.1016/j.reprotox.2007.05.010

[7] Baccarelli A , Bollati V. Epigenetics and environmental chemicals. *Curr Opin Pediatr* 2009;21:243–51. 19663042 10.1097/mop.0b013e32832925cc PMC3035853

[8] Medvedeva YA , Khamis AM, Kulakovskiy IV et al. Effects of cytosine methylation on transcription factor binding sites. *BMC Genomics* 2014;15:119. 10.1186/1471-2164-15-119 PMC3986887 24669864

[9] Yang AS , Estécio MR, Doshi K et al. A simple method for estimating global DNA methylation using bisulfite PCR of repetitive DNA elements. *Nucleic Acids Res* 2004;32:e38. 10.1093/nar/gnh032 PMC373427 14973332

[10] Nelson HH , Marsit CJ, Kelsey KT. Global methylation in exposure biology and translational medical science. *Environ Health Perspect* 2011;119:1528–33. 21669556 10.1289/ehp.1103423 PMC3226501

[11] Barchitta M , Quattrocchi A, Maugeri A et al. LINE-1 hypomethylation in blood and tissue samples as an epigenetic marker for cancer risk: a systematic review and meta-analysis. *PLoS One* 2014;9:e109478. 10.1371/journal.pone.0109478 PMC4183594 25275447

[12] Kazazian HH , Goodier JL. LINE drive. Retrotransposition and genome instability. *Cell* 2002;110:277–80. 12176313 10.1016/s0092-8674(02)00868-1

[13] Bartel DP . MicroRNAs: target recognition and regulatory functions. *Cell* 2009;136:215–33. 19167326 10.1016/j.cell.2009.01.002 PMC3794896

[14] Chuang JC , Jones PA. Epigenetics and microRNAs. *Pediatr Res* 2007;61:24r–9r. 10.1203/pdr.0b013e3180457684 17413852

[15] Malumbres M . miRNAs and cancer: an epigenetics view. *Mol Aspects Med* 2013;34:863–74. 22771542 10.1016/j.mam.2012.06.005 PMC5791883

[16] Lu J , Getz G, Miska EA et al. MicroRNA expression profiles classify human cancers. *Nature* 2005;435:834–8. 15944708 10.1038/nature03702

[17] Condrat CE , Thompson DC, Barbu MG et al. miRNAs as biomarkers in disease: latest findings regarding their role in diagnosis and prognosis. *Cells* 2020;9:276. doi: 10.3390/cells9020276. PMC7072450 31979244

[18] Harrill AH , McCullough SD, Wood CE et al. MicroRNA biomarkers of toxicity in biological matrices. *Toxicol Sci* 2016;152:264–72. 27462126 10.1093/toxsci/kfw090

[19] Farahani M , Rezaei-Tavirani M, Arjmand B. A systematic review of microRNA expression studies with exposure to bisphenol A. *J Appl Toxicol* 2021;41:4–19. 32662106 10.1002/jat.4025

[20] Liu R , Kong W, Zheng S et al. Prognostic significance of microRNA miR-24 in cancers: a meta-analysis. *Bioengineered* 2021;12:450–60. 33550881 10.1080/21655979.2021.1875662 PMC8291878

[21] Pan LJ , Wang X, Ling Y et al. MiR-24 alleviates cardiomyocyte apoptosis after myocardial infarction via targeting BIM. *Eur Rev Med Pharmacol Sci* 2020;24:7549. 10.26355/eurrev_202007_22191 32744654

[22] Xu M , Palmer AK, Ding H et al. Targeting senescent cells enhances adipogenesis and metabolic function in old age. *Elife* 2015;4:e12997. 10.7554/eLife.12997 PMC4758946 26687007

[23] Xu L , Chen Z, Xue F et al. MicroRNA-24 inhibits growth, induces apoptosis, and reverses radioresistance in laryngeal squamous cell carcinoma by targeting X-linked inhibitor of apoptosis protein. *Cancer Cell Int* 2015;15:61. 10.1186/s12935-015-0217-x PMC4477309 26106283

[24] De Felice B , Manfellotto F, Palumbo A et al. Genome-wide microRNA expression profiling in placentas from pregnant women exposed to BPA. *BMC Med Genomics* 2015;8:56. 10.1186/s12920-015-0131-z PMC4562201 26345457

[25] Miao M , Zhou X, Li Y et al. LINE-1 hypomethylation in spermatozoa is associated with bisphenol A exposure. *Andrology* 2014;2:138–44. 24293158 10.1111/j.2047-2927.2013.00166.x

[26] Tubio JMC , Li Y, Ju YS et al. Mobile DNA in cancer. Extensive transduction of nonrepetitive DNA mediated by L1 retrotransposition in cancer genomes. *Science* 2014;345:1251343. 10.1126/science.1251343 PMC4380235 25082706

[27] Wang X , Wei Y, Guo T et al. Epigenetic effect of long-term bisphenol A exposure on human breast adenocarcinoma cells. *Toxicol Environ Chem* 2018;100:258–66.

[28] Li C-C , Wang Z-Y, Wang L-J et al. Biosensors for epigenetic biomarkers detection: a review. *Biosens Bioelectron* 2019;144:111695. 10.1016/j.bios.2019.111695 31526982

[29] EFSA . Scientific opinion on the risks to public health related to the presence of bisphenol A (BPA) in foodstuffs: executive summary. *EFSA J* 2015;3978:1–23.

[30] ECHA . *Candidate List of substances of very high concern for Authorisation (published in accordance with Article 59(10) of the REACH Regulation): 4,4ʹ-isopropylidenediphenol*. https://emea01.safelinks.protection.outlook.com/?url=https%3A%2F%2Fecha.europa.eu%2Fcandidate-list-table%3Fp_p_id%3Ddisslists_WAR_disslistsportlet%26p_p_lifecycle%3D1%26p_p_state%3Dnormal%26p_p_mode%3Dview%26_disslists_WAR_disslistsportlet_javax.portlet.action%3DsearchDissLists&amp;data=04%7C01%7C%7Cfef2ee1386c24a52fc8108d99dd96bcf%7C84df9e7fe9f640afb435aaaaaaaaaaaa%7C1%7C0%7C637714379416264127%7CUnknown%7CTWFpbGZsb3d8eyJWIjoiMC4wLjAwMDAiLCJQIjoiV2luMzIiLCJBTiI6Ik1haWwiLCJXVCI6Mn0%3D%7C1000&sdata?m76VR34R%2FkHke0aQD6QRthHnya%2BeqTpvEXstuQAUcus%3D&amp;reserved=0 (2 November 2021, date last accessed). ECHA European Chemical Agency.

[31] Rochester JR , Bolden AL. Bisphenol S and F: a systematic review and comparison of the hormonal activity of bisphenol A substitutes. *Environ Health Perspect* 2015;123:643–50. 25775505 10.1289/ehp.1408989 PMC4492270

[32] Wu P , Cai Z, Jin H et al. Adsorption mechanisms of five bisphenol analogues on PVC microplastics. *Sci Total Environ* 2019;650:671–8. 30212696 10.1016/j.scitotenv.2018.09.049

[33] Zhou X , Wei J, Liu K et al. Adsorption of bisphenol A based on synergy between hydrogen bonding and hydrophobic interaction. *Langmuir* 2014;30:13861–8. 25365708 10.1021/la502816m

[34] Das MK , Andreassen R, Haugen TB et al. Identification of endogenous controls for use in miRNA quantification in human cancer cell lines. *Cancer Genomics Proteomics* 2016;13:63–8. 26708600

[35] Korma W , Mihret A, Tarekegn A et al. Identification of circulating miR-22-3p and miR-93-5p as stable endogenous control in tuberculosis study. *Diagnostics (Basel)* 2020;10:868. doi: 10.3390/diagnostics10110868. PMC7690830 33114169

[36] Jedeon K , De la Dure-molla M, Brookes SJ et al. Enamel defects reflect perinatal exposure to bisphenol A. *Am J Pathol* 2013;183:108–18. 23764278 10.1016/j.ajpath.2013.04.004 PMC3703547

[37] Valvi D , Casas M, Mendez MA et al. Prenatal bisphenol a urine concentrations and early rapid growth and overweight risk in the offspring. *Epidemiology* 2013;24:791–9. 24036610 10.1097/EDE.0b013e3182a67822

[38] Weng YI , Hsu PY, Liyanarachchi S et al. Epigenetic influences of low-dose bisphenol A in primary human breast epithelial cells. *Toxicol Appl Pharmacol* 2010;248:111–21. 20678512 10.1016/j.taap.2010.07.014 PMC2946518

[39] Vandenberg LN , Colborn T, Hayes TB et al. Hormones and endocrine-disrupting chemicals: low-dose effects and nonmonotonic dose responses. *Endocr Rev* 2012;33:378–455. 22419778 10.1210/er.2011-1050 PMC3365860

[40] Völkel W , Colnot T, Csanády GA et al. Metabolism and kinetics of bisphenol a in humans at low doses following oral administration. *Chem Res Toxicol* 2002;15:1281–7. 12387626 10.1021/tx025548t

[41] Matthews JB , Twomey K, Zacharewski TR. In vitro and in vivo interactions of bisphenol A and its metabolite, bisphenol A glucuronide, with estrogen receptors alpha and beta. *Chem Res Toxicol* 2001;14:149–57. 11258963 10.1021/tx0001833

[42] Sargis RM , Johnson DN, Choudhury RA et al. Environmental endocrine disruptors promote adipogenesis in the 3T3-L1 cell line through glucocorticoid receptor activation. *Obesity (Silver Spring)* 2010;18:1283–8. 19927138 10.1038/oby.2009.419 PMC3957336

[43] Liao C , Kannan K. Determination of free and conjugated forms of bisphenol A in human urine and serum by liquid chromatography-tandem mass spectrometry. *Environ Sci Technol* 2012;46:5003–9. 22489688 10.1021/es300115a

[44] Boucher JG , Boudreau A, Ahmed S et al. In vitro effects of bisphenol A β-d-Glucuronide (BPA-G) on adipogenesis in human and murine preadipocytes. *Environ Health Perspect* 2015;123:1287–93. 26018136 10.1289/ehp.1409143 PMC4671229

[45] Veiga-Lopez A , Luense LJ, Christenson LK et al. Developmental programming: gestational bisphenol-A treatment alters trajectory of fetal ovarian gene expression. *Endocrinology* 2013;154:1873–84. 23525218 10.1210/en.2012-2129 PMC3628019

[46] Mahesh G , Biswas R. MicroRNA-155: a master regulator of inflammation. *J Interferon Cytokine Res* 2019;39:321–30. 30998423 10.1089/jir.2018.0155 PMC6591773

[47] Liu D , Hu Y, Wang J et al. WITHDRAWN: bisphenol S triggers the malignancy of hemangioma cells via regulation of basic fibroblast growth factor. *Chem Biol Interact* 2020;315:108866. 10.1016/j.cbi.2019.108866 31669319

[48] Sabry R , Saleh AC, Stalker L et al. Effects of bisphenol A and bisphenol S on microRNA expression during bovine (*B**os taurus*) oocyte maturation and early embryo development. *Reprod Toxicol* 2021;99:96–108. 33285269 10.1016/j.reprotox.2020.12.001

[49] Avissar-Whiting M , Veiga KR, Uhl KM et al. Bisphenol A exposure leads to specific microRNA alterations in placental cells. *Reprod Toxicol* 2010;29:401–6. 20417706 10.1016/j.reprotox.2010.04.004 PMC2896875

[50] Esmaili MA , Kazemi A, Zaker F et al. Effects of reduced Mir-24 expression on plasma methotrexate levels, therapy-related toxicities, and patient outcomes in pediatric acute lymphoblastic leukemia. *Rep Biochem Mol Biol* 2020;8:358–65. 32582793 PMC7275838

[51] Cho H , Kim SJ, Park H-W et al. A relationship between miRNA and gene expression in the mouse Sertoli cell line after exposure to bisphenol A. *BioChip J* 2010;4:75–81.

[52] Gao GZ , Zhao Y, Li HX et al. A-elicited miR-146a-5p impairs murine testicular steroidogenesis through negative regulation of Mta3 signaling. *Biochem Biophys Res Commun* 2018;501:478–85. 29746863 10.1016/j.bbrc.2018.05.017

[53] Dong X , Zhang Z, Meng S et al. Parental exposure to bisphenol A and its analogs influences zebrafish offspring immunity. *Sci Total Environ* 2018;610–611:291–7. doi: 10.1016/j.scitotenv.2017.08.057. PMC7112096 28806546

[54] Zhang J , Ji X, Zhou D et al. miR-143 is critical for the formation of primordial follicles in mice. *Front Biosci (Landmark Ed)* 2013;18:588–97. 23276944 10.2741/4122

[55] Xie X , Song J, Li G. MiR-21a-5p suppresses bisphenol A-induced pre-adipocyte differentiation by targeting map2k3 through MKK3/p38/MAPK. *Biochem Biophys Res Commun* 2016;473:140–6. 26996129 10.1016/j.bbrc.2016.03.066

[56] Tilghman SL , Bratton MR, Segar HC et al. Endocrine disruptor regulation of microRNA expression in breast carcinoma cells. *PLoS One* 2012;7:e32754. 10.1371/journal.pone.0032754 PMC3293845 22403704

[57] Meng F , Henson R, Wehbe-Janek H et al. MicroRNA-21 regulates expression of the PTEN tumor suppressor gene in human hepatocellular cancer. *Gastroenterology* 2007;133:647–58. 17681183 10.1053/j.gastro.2007.05.022 PMC4285346

[58] Sauer SJ , Tarpley M, Shah I et al. Bisphenol A activates EGFR and ERK promoting proliferation, tumor spheroid formation and resistance to EGFR pathway inhibition in estrogen receptor-negative inflammatory breast cancer cells. *Carcinogenesis* 2017;38:252–60. 28426875 10.1093/carcin/bgx003 PMC5963742

[59] Hui L , Li H, Lu G et al. Low dose of bisphenol A modulates ovarian cancer gene expression profile and promotes epithelial to mesenchymal transition via canonical Wnt pathway. *Toxicol Sci* 2018;164:527–38. 29718440 10.1093/toxsci/kfy107

[60] Wang Z . ErbB receptors and cancer. *Methods Mol Biol* 2017;1652:3–35. 28791631 10.1007/978-1-4939-7219-7_1

[61] Lei B , Xu L, Tang Q et al. Molecular mechanism study of BPAF-induced proliferation of ERα-negative SKBR-3 human breast cancer cells in vitro/in vivo. *Sci Total Environ* 2021;775:145814. 10.1016/j.scitotenv.2021.145814 33621883

[62] Lei B , Tang Q, Sun S et al. Insight into the mechanism of tetrachlorobisphenol A (TCBPA)-induced proliferation of breast cancer cells by GPER-mediated signaling pathways. *Environ Pollut* 2021;275:116636. 10.1016/j.envpol.2021.116636 33582643

[63] Ma Z , Parris AB, Howard EW et al. In utero exposure to bisphenol a promotes mammary tumor risk in MMTV-Erbb2 transgenic mice through the induction of ER-erbB2 crosstalk. *Int J Mol Sci* 2020;21:9. 10.3390/ijms21093095 PMC7247154 32353937

[64] Wee P , Wang Z. Epidermal growth factor receptor cell proliferation signaling pathways. *Cancers (Basel)* 2017;9:5. 10.3390/cancers9050052 PMC5447962 28513565

[65] Wang K , Qiu L, Zhu J et al. Environmental contaminant BPA causes intestinal damage by disrupting cellular repair and injury homeostasis in vivo and in vitro. *Biomed Pharmacother* 2021;137:111270. 10.1016/j.biopha.2021.111270 33485121

[66] Awada Z , Nasr R, Akika R et al. DNA methylome-wide alterations associated with estrogen receptor-dependent effects of bisphenols in breast cancer. *Clin Epigenet* 2019;11:138. 10.1186/s13148-019-0725-y PMC6785895 31601247

[67] Nahar MS , Liao C, Kannan K et al. In utero bisphenol A concentration, metabolism, and global DNA methylation across matched placenta, kidney, and liver in the human fetus. *Chemosphere* 2015;124:54–60. 25434263 10.1016/j.chemosphere.2014.10.071 PMC4297568

