# Supplementary material for: Different bisphenols induce non-monotonous changes in miRNA expression and LINE-1 methylation in two cell lines
Source: Environ Epigenet. 2021 Nov 25;7(1):dvab011. doi: 10.1093/eep/dvab011 (PMC8633614; doi:10.1093/eep/dvab011)
Supplement: dvab011_Supp [file dvab011_supp.zip › suppl data.docx]

| Caco-2 cells | Relative fold changes and standard deviation of the respective bisphenol treatment  (in relation to a control value of 1 of the untreated control) | | | | |
| --- | --- | --- | --- | --- | --- |
|  | **0.001 µg/ml** | **0.01 µg/ml** | **0.1 µg/ml** | **1 µg/ml** | **10 µg/ml** |
|  |  |  |  |  |  |
| Bisphenol A |  |  |  |  |  |
| \| miR-24 \| \| --- \| \| miR-155 \| \| miR-21 \| \| miR-146 \| | \| 0.822 ± 0.199 \| \| --- \| \| 0.882 ± 0.237 \| \| 0.751 ± 0.103 \| \| 0.799 ± 0.220 \| | \| 0.874 ± 0.071 \| \| --- \| \| 0.856 ± 0.650 \| \| 0.846 ± 0.213 \| \| 1.164 ± 0.066 \| | \| 0.947 ± 0.198 \| \| --- \| \| 0.527 ± 0.218 \| \| 1.017 ± 0.175 \| \| **1.808 ± 0.070***** \| | \| 1.231 ± 0.085 \| \| --- \| \| 1.173 ± 0.096 \| \| 0.885 ± 0.035 \| \| **1.476 ± 0.216**** \| | \| 1.131 ± 0.276 \| \| --- \| \| 1.052 ± 0.306 \| \| 0.970 ± 0.072 \| \| 1.159 ± 0.112 \| |
|  |  |  |  |  |  |
| BPA ß-D-Glucuronide |  |  |  |  |  |
| \| miR-24 \| \| --- \| \| miR-155 \| \| miR-21 \| \| miR-146 \| | \| 1.027 ± 0.010 \| \| --- \| \| 0.826 ± 0.113 \| \| 0.833 ± 0.116 \| \| 0.909 ± 0.018 \| | \| 0.831 ± 0.131 \| \| --- \| \| 0.600 ± 0.464 \| \| 0.918 ± 0.074 \| \| 0.848 ± 0.146 \| | \| 0.720 ± 0.248 \| \| --- \| \| 0.748 ± 0.261 \| \| 0.897 ± 0.080 \| \| 1.062 ± 0.105 \| | \| 0.797 ± 0.133 \| \| --- \| \| 1.194 ± 0.556 \| \| 0.837 ± 0.076 \| \| **0.824 ± 0.037*** \| | \| 0.899 ± 0.088 \| \| --- \| \| 1.440 ± 0.111 \| \| 0.878 ± 0.070 \| \| 0.946 ± 0.060 \| |
|  |  |  |  |  |  |
| Bisphenol S |  |  |  |  |  |
| \| miR-24 \| \| --- \| \| miR-155 \| \| miR-21 \| \| miR-146 \| | \| 0.930 ± 0.262 \| \| --- \| \| 0.720 ± 0.213 \| \| 0.890 ± 0.120 \| \| 0.972 ± 0.098 \| | \| 0.823 ± 0.080 \| \| --- \| \| 0.787 ± 0.173 \| \| 0.911 ± 0.093 \| \| 0.987 ± 0.059 \| | \| 0.765 ± 0.163 \| \| --- \| \| 0.673 ± 0.156 \| \| **0.703 ± 0.103*** \| \| 0.897 ± 0.287 \| | \| 1.114 ± 0.178 \| \| --- \| \| **0.506 ± 0.053*** \| \| 0.791 ± 0.127 \| \| 1.154 ± 0.070 \| | \| 0.829 ± 0.047 \| \| --- \| \| 0.858 ± 0.192 \| \| **0.497 ± 0.161***** \| \| 0.856 ± 0.092 \| |
|  |  |  |  |  |  |
| Bisphenol F |  |  |  |  |  |
| \| miR-24 \| \| --- \| \| miR-155 \| \| miR-21 \| \| miR-146 \| | \| 0.988 ± 0.129 \| \| --- \| \| 0.649 ± 0.006 \| \| 0.964 ± 0.185 \| \| 0.820 ± 0.228 \| | \| 0.797 ± 0.124 \| \| --- \| \| 0.656 ± 0.120 \| \| 0.952 ± 0.100 \| \| 1.021 ± 0.161 \| | \| **0.604 ± 0.198*** \| \| --- \| \| **0.559 ± 0.412*** \| \| **0.720 ± 0.056*** \| \| 0.843 ± 0.164 \| | \| 0.769 ± 0.176 \| \| --- \| \| 0.677 ± 0.134 \| \| 0.877 ± 0.112 \| \| 0.885 ± 0.088 \| | \| 0.871 ± 0.154 \| \| --- \| \| **0.376 ± 0.110**** \| \| 0.850 ± 0.081 \| \| 0.862 ± 0.061 \| |
|  |  |  |  |  |  |
| p,p'-Oxybisphenol |  |  |  |  |  |
| \| miR-24 \| \| --- \| \| miR-155 \| \| miR-21 \| \| miR-146 \| | \| **0.244 ± 0.136****** \| \| --- \| \| 1.640 ± 0.338 \| \| 0.922 ± 0.298 \| \| 0.896 ± 0.164 \| | \| 1.031 ± 0.170 \| \| --- \| \| 0.763 ± 0.545 \| \| 1.255 ± 0.206 \| \| 0.815 ± 0.074 \| | \| **0.656 ± 0.082**** \| \| --- \| \| 0.467 ± 0.038 \| \| 1.039 ± 0.249 \| \| 0.875 ± 0.189 \| | \| 0.843 ± 0.070 \| \| --- \| \| 0.819 ± 0.608 \| \| 0.842 ± 0.231 \| \| 0.737 ± 0.195 \| | \| 0.895 ± 0.096 \| \| --- \| \| 1.097 ± 0.142 \| \| 0.764 ± 0.215 \| \| 0.827 ± 0.136 \| |
|  |  |  |  |  |  |

Data are shown as the mean of relative fold changes ± SD of three replicates per treatment.

*: p < 0.05; **: p < 0.01; ***: p < 0.001; and ****: p < 0.0001 compared to control

| Human Lung Fibroblasts | Relative fold changes and standard deviation of the respective bisphenol treatment  (in relation to a control value of 1 of the untreated control) | | | | |
| --- | --- | --- | --- | --- | --- |
|  | **0.001 µg/ml** | **0.01 µg/ml** | **0.1 µg/ml** | **1 µg/ml** | **10 µg/ml** |
|  |  |  |  |  |  |
| Bisphenol A |  |  |  |  |  |
| \| miR-24 \| \| --- \| \| miR-155 \| \| miR-21 \| \| miR-146 \| | \| 1.017 ± 0.168 \| \| --- \| \| 0.950 ± 0.284 \| \| 0.783 ± 0.276 \| \| 0.988 ± 0.313 \| | \| 0.781 ± 0.254 \| \| --- \| \| 1.165 ± 0.167 \| \| 0.980 ± 0.188 \| \| 1.365 ± 0.321 \| | \| 1.033 ± 0.232 \| \| --- \| \| 1.215 ± 0.504 \| \| 0.948 ± 0.416 \| \| 1.358 ± 0.389 \| | **2.019 ± 0.415*****  **1.746 ± 0.421***  **1.975 ± 0.498****  **1.947 ± 0.524**** | \| 0.843 ± 0.012 \| \| --- \| \| 0.758 ± 0.114 \| \| 0.903 ± 0.326 \| \| 0.773 ± 0.192 \| |
|  |  |  |  |  |  |
| BPA ß-D-Glucuronide |  |  |  |  |  |
| \| miR-24 \| \| --- \| \| miR-155 \| \| miR-21 \| \| miR-146 \| | \| \| 0.918 ± 0.047 \| \| --- \| \| 0.633 ± 0.056 \| \| **0.779 ± 0.159*** \| \| 1.091 ± 0.086 \| \| \| --- \| --- \| --- \| --- \| --- \| | \| \| 0.830 ± 0.118 \| \| --- \| \| 0.840 ± 0.272 \| \| **0.746 ± 0.056*** \| \| 0.973 ± 0.225 \| \| \| --- \| --- \| --- \| --- \| --- \| | \| 1.141 ± 0.218 \| \| --- \| \| **0.542 ± 0.109*** \| \| **0.750 ± 0.040*** \| \| 0.868 ± 0.068 \| | \| 1.221 ± 0.167 \| \| --- \| \| 0.636 ± 0.063 \| \| 0.906 ± 0.130 \| \| 0.925 ± 0.062 \| | \| 1.190 ± 0.101 \| \| --- \| \| 0.875 ± 0.333 \| \| **0.695 ± 0.069**** \| \| 0.975 ± 0.122 \| |
|  |  |  |  |  |  |
| Bisphenol S |  |  |  |  |  |
| \| miR-24 \| \| --- \| \| miR-155 \| \| miR-21 \| \| miR-146 \| | \| 1.119 ± 0.073 \| \| --- \| \| 0.913 ± 0.088 \| \| 0.821 ± 0.141 \| \| 1.060 ± 0.335 \| | \| 1.216 ± 0.240 \| \| --- \| \| 1.226 ± 0.471 \| \| 0.777 ± 0.081 \| \| 1.099 ± 0.051 \| | \| 1.187 ± 0.047 \| \| --- \| \| 0.697 ± 0.174 \| \| 0.821 ± 0.191 \| \| 0.967 ± 0.152 \| | \| **1.620 ± 0.547*** \| \| --- \| \| 0.727 ± 0.132 \| \| 0.957 ± 0.178 \| \| 0.836 ± 0.011 \| | \| 0.898 ± 0.167 \| \| --- \| \| 1.883 ± 0.852 \| \| 0.893 ± 0.328 \| \| **1.818 ± 0.508**** \| |
|  |  |  |  |  |  |
| Bisphenol F |  |  |  |  |  |
| \| miR-24 \| \| --- \| \| miR-155 \| \| miR-21 \| \| miR-146 \| | \| 1.173 ± 0.137 \| \| --- \| \| 1.246 ± 0.271 \| \| 1.070 ± 0.172 \| \| 1.108 ± 0.032 \| | \| 1.264 ± 0.338 \| \| --- \| \| 1.050 ± 0.339 \| \| **0.612 ± 0.160**** \| \| 1.184 ± 0.155 \| | \| 1.134 ± 0.016 \| \| --- \| \| 0.789 ± 0.122 \| \| 0.802 ± 0.078 \| \| 0.950 ± 0.032 \| | \| 0.962 ± 0.122 \| \| --- \| \| 0.704 ± 0.105 \| \| **0.609 ± 0.082**** \| \| 0.760 ± 0.112 \| | \| 0.851 ± 0.191 \| \| --- \| \| 0.707 ± 0.135 \| \| **0.590 ± 0.069**** \| \| 0.756 ± 0.211 \| |
|  |  |  |  |  |  |
| p,p'-Oxybisphenol |  |  |  |  |  |
| \| miR-24 \| \| --- \| \| miR-155 \| \| miR-21 \| \| miR-146 \| | \| 1.172 ± 0.215 \| \| --- \| \| 0.976 ± 0.201 \| \| **0.690 ± 0.188**** \| \| 1.096 ± 0.166 \| | \| 1.096 ± 0.153 \| \| --- \| \| 0.873 ± 0.263 \| \| 0.767 ± 0.177 \| \| 0.905 ± 0.143 \| | \| 1.096 ± 0.219 \| \| --- \| \| 0.769 ± 0.103 \| \| 0.912 ± 0.150 \| \| 0.906 ± 0.073 \| | \| **1.561 ± 0.208**** \| \| --- \| \| 0.898 ± 0.214 \| \| 0.909 ± 0.152 \| \| 1.134 ± 0.142 \| | \| 0.947 ± 0.145 \| \| --- \| \| 0.668 ± 0.154 \| \| 0.725 ± 0.049 \| \| **0.707 ± 0.155**** \| |
|  |  |  |  |  |  |

Data are shown as the mean of relative fold changes ± SD of three replicates per treatment.

*: p < 0.05; **: p < 0.01; ***: p < 0.001; and ****: p < 0.0001 compared to control.
